# Impact of Nicotine Replacement Therapy on Post-Cessation Mood Profile by Pre-Cessation Depressive Symptoms

**DOI:** 10.1186/1617-9625-3-2-17

**Published:** 2006-08-15

**Authors:** Tellervo Korhonen, Taru H Kinnunen, Arthur J Garvey

**Affiliations:** 1 Tobacco Dependence Treatment and Research, Harvard School of Dental Medicine, Boston, MA; 2 Smoking Cessation Research Program, Harvard School of Dental Medicine, Boston, MA; 3 Department of Public Health, University of Helsinki, Finland; 4 This study was conducted within the Harvard School of Dental Medicine, Boston, USA

## Abstract

We evaluated the effects of Nicotine Replacement Therapy (NRT) on the Profile of Mood States (POMS), testing whether pre-cessation depressive symptoms modify NRT's effects on POMS. Out of 608 smokers attempting to quit with NRT, this secondary analysis included 242 participants abstinent for at least two weeks. We measured pre-cessation depressive symptoms with the Center for Epidemiological Studies Depression Scale. At 1, 7, and 14 post-cessation days we examined 6 self-reported POMS, i.e. feeling 'anxious', 'sad', 'confused', 'angry', 'energetic' and 'fatigue'. The results of the ANCOVA models suggested no NRT effects on feeling anxious, energetic or fatigue. We found that pre-cessation depression modified NRT effects in some specific mood states, such as depression by NRT- interaction effects on feeling confused and feeling angry. On average, the depressed participants in the placebo groups had the highest symptom scores. However, those depressed in NRT conditions did not have significantly higher symptom scores compared to the non-depressed groups. In treating those negative moods NRT may be particularly important for persons with depressive symptoms before cessation.

## Introduction

Prolonged tobacco use triples mortality rates, whereas smoking cessation halves or even avoids those hazards [1]. According to the PHS Clinical Practice Guidelines [2] two main treatments for tobacco dependence are behavioural counselling and pharmacotherapy. Six first-line pharmaco-therapies have been identified that increase smoking abstinence, such as five forms of nicotine replacement therapy (NRT), including nicotine gum, inhaler, nasal spray, patch, and lozenge, and non-nicotine bupropion SR.

Most smokers would like to stop smoking, but for many of them it is very difficult because of withdrawal symptoms [2]. According to the DSM-IV diagnostic criteria for nicotine withdrawal [3], the symptoms can appear within two hours after the last use of tobacco, and usually last from a few days to four weeks. Among the withdrawal signs several measurable symptoms have been identified, i.e. depressed mood, insomnia, irritability, anxiety, difficulty concentrating, restlessness, decreased heart rate, weight gain and craving [4]. These withdrawal signs may be conceptualized as cognitive, affective, somatic, and craving, although they are not mutually exclusive. For example, difficulty concentrating and impaired cognitive performance are identified as part of the nicotine withdrawal syndrome [3].

Further, mood related withdrawal symptoms, such as depressed mood, anxiety and irritability, remain major impediments for smoking cessation, early relapse being associated with increase in negative mood [5]. Although not directly measuring the withdrawal symptomatology as listed in the DSM-IV diagnostic criteria for nicotine withdrawal [3], the Profile of Mood States (POMS) [6] can be used to track most nicotine withdrawal signs.

According to Ward et al. [7], the POMS, including three affective (feeling anxious, sad, angry), one cognitive (feeling confused) and two somatic energy-related (feeling energetic, fatigue) states of mood, can be identified as transient abstinence effects during the first month after cessation.

The efficacy of NRT for smoking cessation is well established [2]. One of mechanisms by which NRT may work is by reducing affective, cognitive and somatic symptoms during abstinence from smoking [4]. However, some studies have observed only limited effects [8,9], whereas some studies suggest that NRT has a favourable impact on negative mood and facilitate attention [10,11].

Smokers with psychiatric co-morbidities, including depressive symptoms, have been mentioned as a special subgroup requiring additional research within efficacy of tobacco dependence treatments [2]. Also, there has been substantial variability in treatment response among various subgroups of quitters, such as smokers with co-morbidities [4]. These recent developments challenge to analyze more specifically how NRT works in those subgroups.

Concerning depressive symptoms, some recent studies have suggested that a history of lifetime depression would not be a risk factor for smoking relapses [12,13]. However, current depressive symptoms are suggested to be impediments for quitting and smoking may function as a self-medicating device for those with such symptoms [14,15].

Also, currently depressed individuals smoke more frequently and are more dependent than non-depressed [16-18]. Further, smokers with current depression have lower cessation rates than non-depressed [18,19] and smoking cessation may elicit depression among patients with prior affective disorder [17,20,21]. Concerning NRT efficacy for depressed smokers, the effects of nicotine gum on abstinence have been found particularly beneficial among the depressed quitters [22].

However, there has been little evidence on effects of nicotine treatment on mood, such as POMS, among participants with Major Depressive Disorder [23]. Previous research raises a further question whether pre-cessation depressive symptoms modify NRT effects on post-cessation mood during the abstinence, i.e. if there is interaction between treatment and pre-cessation depressive symptoms. Following these challenges, the aim of this study was to investigate whether pre-cessation depressive symptoms modify NRT effects on post cessation mood states.

## Methods

### Sample and Procedures

The current data are from a randomized controlled nicotine gum trial that took place during the period 1992–1996 in Boston, MA, USA. The original sample included 608 smokers attempting to quit. Before their quit days, participants were randomly assigned into a NRT (2 or 4 mg nicotine gum) or placebo (0 mg placebo gum) condition. All participants were provided brief counselling. Details of the trial procedures and main results have been previously reported [24].

**Figure 1:**
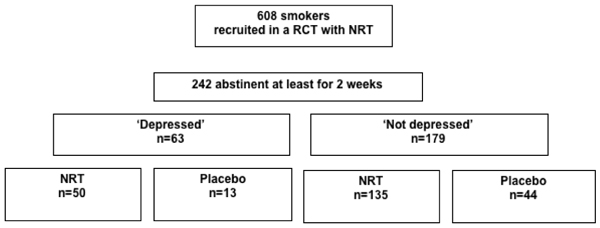
**The flow chart of the studied subgroups**.

The current study is a secondary analysis including 242 participants who remained abstinent for at least 2 weeks (39.8% of the original sample). Self-reported abstinence was verified by carbon monoxide (CO) measurements. Relapse in this study was defined as a return to a regular pattern of smoking, i.e. seven or more consecutive days or episodes of smoking [25]. Figure 1 shows the flow chart of the studied subgroups by depression and treatment condition analyzed in this study.

### Measures

In order to categorize our sub-sample into "depressed' and 'non-depressed' participants before quitting smoking we used the data collected at the baseline visit. To measure pre-cessation depressive symptoms we used the Centre for Epidemiological Studies Depression Scale (CES-D) [26,27]. It is a widely used self-report instrument including 20 items on frequency and severity of given symptoms a person had experienced during the past week. Four items were first reverse-scored and then summed to create a depression score (ranging from 0 to 60). To be classified as 'depressed', participants had to have a CES-D score of at least 16; to be categorized as 'non depressed', participants had to have a CES-D score of less than 16. The cutoff of 16 is considered a standard for current depression, although somewhat higher scores have been suggested as well [28]. However, labelling those with low CES-D score as 'non depressed' and those with high CES-D score as 'depressed' was used more for brevity through the paper rather than for referring to depression diagnosis.

As proxy for withdrawal symptoms we investigated six mood-related symptoms at 1, 7 and 14 post-cessation days. These self-reported measures were adopted from the Profile of Mood States (POMS) [6] to examine four affective and cognitive symptoms: 'feeling anxious', 'feeling sad', 'feeling confused' and 'feeling angry', as well as two energy-related symptoms: 'feeling energetic' and 'feeling fatigue'. The actual questions for each item were worded as follows: "How anxious/sad or depressed/confused/angry/energetic/fatigued have you felt during the past 24 hours?" Possible responses for each item ranged from 0 (not at all) to 4 (extremely). Similar measures for subjective mood related withdrawal symptoms during smoking cessation have been successfully applied in an earlier analysis of the same research study [24].

### Statistical Analyses

For descriptive analyses we employed *Chi-Square *tests for categorical variables and *t *tests for age, Fagerström Test of Nicotine Dependence (FTND), POMS and CES-D scores. To assess the effects of NRT and depression on the post-cessation mood symptoms we conducted a set of mixed-design repeated measures analyses of covariance (ANCOVA) for each POMS variable. The main models included all main effects, i.e. depression, NRT and time, the baseline POMS measure as a covariate, plus all interactions. For all POMS the scores were computed separately among the participants scoring high ('depressed') and low ('non-depressed') in the pre-cessation depressive symptoms. The results of covariance analysis models give estimates how significant, on average, each factor, i.e. NRT, depression, time, or any interaction, is in the model. Further, in order to identify significant pair-wise differences at specific time points, i.e. at 1, 7, and14 days, we computed the Tukey HSD Post Hoc tests, which are suitable and reliable tests for multiple tests between may subsamples [29]. Analyses were performed using SAS v. 8.02 for Windows and Statistica v. 6.1 for Windows.

## Results

### Drop-out Analysis

Table 1 shows comparison of selected baseline characteristics between the participants included in this analysis (i.e. abstinent for at least 2 weeks) and those who were excluded (i.e. relapsed before 2 weeks). There were significant differences between abstinent and non abstinent samples in the number of cigarettes per day (*p *= .01), in nicotine dependence measured by the FTND score (*p *= .0001), in feeling sad (*p *= .006) and fatigue (*p *= .003), as well as in baseline depression score (CES-D) (*p *= .003). Those not included scored higher in tobacco consumption and dependence, as well as in baseline moods, such as feeling sad and fatigue and in baseline depression score.

**Table 1:** Baseline characteristics among the participants abstinent for at least 14 days and those abstinent for less than 14 days.

	Abstinent (n = 242)	Non abstinent (n = 366)	*P value*^*a*^
Socio-demographics			
Gender (% of male)	52.1	46.4	.17
Age (years)^b^	41.5 (12.1)	40.4 (11.7)	.24
Race (%)			.27
White	81.4	80.6	
African American	11.6	13.9	
Other	7.0	5.5	
Marital Status (%)			.24
Single	39.7	46.4	
Married	33.5	26.0	
Divorced/separated/widowed	26.8	27.6	
Education (%)			.11
Less than 12 years	3.7	6.0	
12–15 years	50.4	56.3	
16 or more years	45.9	37.7	
Smoking characteristics			
Cigarettes per day	21.7 (10.0)	24.7 (11.7)	.0008
FTND (range 1–10)	5.1 (2.2)	5.8 (2.3)	.0001
POMS ^c^			
Feeling anxious	1.4 (1.1)	1.5 (1.2)	.36
Feeling sad	0.6 (0.8)	0.9 (1.0)	.002
Feeling confused	0.4 (0.8)	0.6 (0.9)	.07
Feeling angry	0.8 (1.0)	0.9 (1.1)	.48
Feeling energetic	2.0 (1.0)	1.8 (1.0)	0.08
Feeling fatigue	1.3 (1.1)	1.6 (1.2)	0.003
Depression score (range 0–60)	11.3 (9.0)	13.7 (10.2)	0.003

^a ^Chi-Square test, except *t *test for age, FTND, POMS and CES-D.

^b ^Values are means with standard deviations in parentheses

^c ^POMS = Profile of Mood Score

### Participant Characteristics

The prevalence of baseline depressive symptoms was 32.2% in the original sample (n = 608), whereas 26.0% among the participants included in this analysis (n = 242) (p < .001). The participant characteristics of the current study by treatment condition are shown in Table 2 separately for the depressed and the non-depressed groups. The only significant difference was in racial categories. Among the depressed group fewer white participants were in the placebo than in the NRT condition (*p *= .05). As shown in Table 2, there were large differences between depressed and non-depressed on most POMS variables at baseline. Thus, all the analyses included baseline score as a covariate.

**Table 2:** Baseline Characteristics by Treatment for the Depressed and Non-depressed among the Participants (n = 242)

	Depressed^a^ (n = 63)			Not Depressed^b^ (n = 179)		
Gender (% of male)	40.0	61.5	.16	53.3	59.1	.50
Age (years)^d^	40.1(11.3)	40.8(6.4)	.85	42.5(12.0)	40.4(11.6)	.32
Race (%)			.05			.97
White	83.7	72.7		82.0	84.6	
African American	12.6	13.6		8.0	7.7	
Other	3.7	13.6		10.0	7.7	
Marital Status (%)			.75			.33
Single	50.0	38.5		35.6	40.9	
Married	24.0	30.8		34.8	40.9	
Divorced/separated/widowed	26.0	30.8		29.6	18.2	
Education (%)			.83			.68
Less than 12 years	4.0	7.7		3.7	2.3	
12–15 years	52.0	53.8		51.1	45.5	
16 or more years	44.0	38.5		45.2	52.3	
Smoking characteristics						
Cigarettes per day	22.7(12.2)	23.2(7.5)	.91	21.9 (9.6)	19.8(6.9)	.22
FTND (range 1–10)	5.6 (2.2)	4.8 (1.9)	.29	5.0 (2.2)	4.7 (2.5)	.39
POMS^e^						
Feeling anxious	1.8 (1.1)	2.0 (1.2)	.61	1.3 (1.1)	1.1 (0.9)	.38
Feeling sad	1.3 (1.0)	1.1 (0.8)	.59	0.3 (0.6)	0.6 (0.9)	.09
Feeling confused	0.9 (1.1)	0.9 (0.9)	.95	0.3 (0.5)	0.2 (0.6)	.93
Feeling angry	1.4 (1.3)	1.5 (1.0)	.91	0.6 (1.0)	0.6 (0.9)	.67
Feeling energetic	1.8 (1.1)	1.7 (0.8)	.66	2.0 (1.0)	2.1 (0.6)	.49
Feeling fatigue	1.8 (1.2)	1.8 (1.0)	.80	1.1 (1.0)	1.3 (1.1)	.17
Depression score (range 0–60)	23.9 (7.2)	23.6(4.9)	.90	6.8 (4.4)	7.3 (4.1)	.54

^a ^CES-D > 15

^b ^CES-D ≤ 15

^c ^Chi-Square test, except t test for age, FTND, POMS and CES-D.

^d ^Values are means with standard deviations in parentheses

^e ^POMS = Profile of Mood Score

### Effects of NRT on POMS

**Figure 2:**
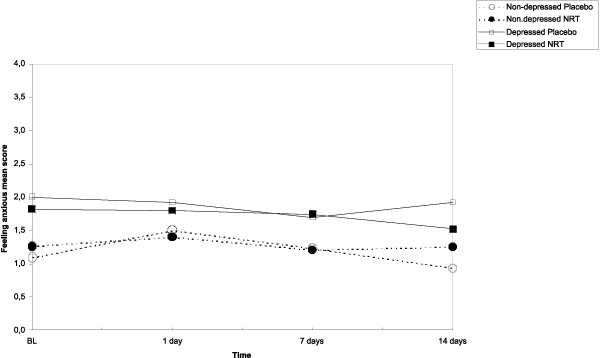
**Average feeling anxious scores by treatment and depression at 1, 7 and 14 days after cessation baseline scores as covariate**.

**Figure 3:**
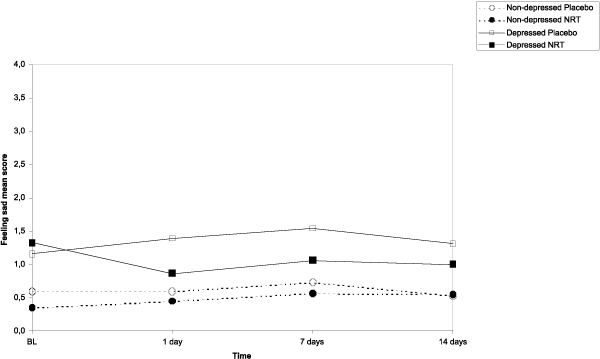
**Average feeling sad scores by treatment and depression at 1, 7 and 14 days after cessation baseline scores as covariate**.

**Figure 4:**
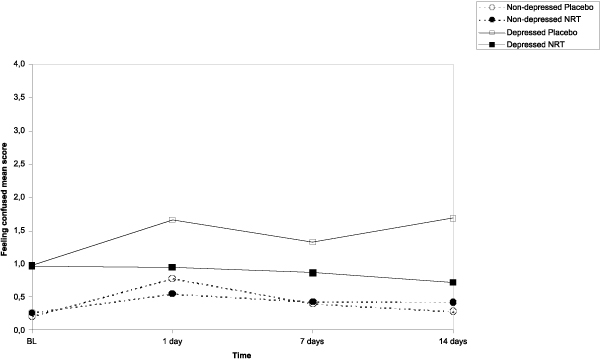
**Average feeling confused scores by treatment and depression at 1, 7 and 14 days after cessation baseline scores as covariate**.

**Figure 5:**
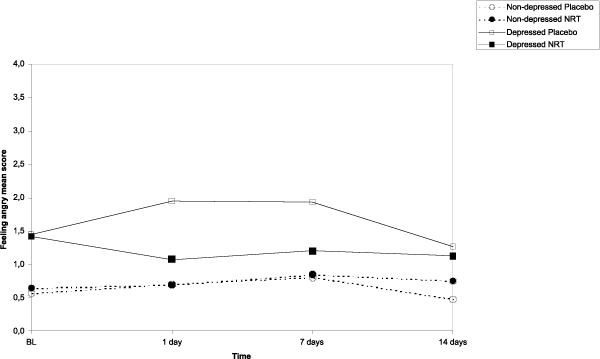
**Average feeling angry scores by treatment and depression at 1, 7 and 14 days after cessation baseline scores as covariate**.

The results of the main effects of NRT, depressive symptoms and time on the post-cessation mood symptoms (feeling anxious, sad, confused, angry, energetic, and fatigue) as well as those interactions are shown in Table 3. Based on the repeated measures ANCOVA analyses, figures 2, 3, 4, 5 show the sets of curves for the observed average POMS scores at baseline, 1, 7 and 14 post-cessation days by the treatment analyses, figures 2, 3, 4, 5 show the sets of curves for the observed average POMS scores at baseline, 1, 7 and 14 post-cessation days by the treatment condition (NRT/Placebo) and by the baseline depressive symptoms status ('non-depressed'/'depressed'), where significant effects were found condition (NRT/Placebo) and by the baseline depressive symptoms status ('non-depressed'/'depressed'), where significant effects were found.

**Table 3:** Results of the repeated measures ANCOVA main models

		*FEELING ANXIOUS*		*FEELING SAD*		*FEELING CONFUSED*		*FEELING ANGRY*		*FEELING ENERGETIC*		*FEELING FATIGUE*	
	df	F	*p*	F	*p*	F	*p*	F	*p*	F	*p*	F	*p*

BASELINE SCORE	1	29.15	< .001	24.80	< .001	42.63	< .001	44.30	< .001	54.50	< .001	23.51	< .001

NRT	1	010	.76	4.48	.03	9.45	.002	3.51	.06	2.81	.09	1.07	.30

CES-D^a^	1	4.78	.03	9.83	.002	14.02	< .001	10.68	.001	0.08	.77	1.88	.17

NRT * CES-D^a^	1	0.22	.64	3.55	.06	8.17	.005	5.47	.02	0.00	.95	1.38	.24

TIME	2	1.10	.33	0.51	.60	1.14	.32	4.16	.02	1.10	.33	0.08	.92

NRT * TIME	2	0.15	.86	0.65	.52	0.44	.65	2.78	.06	0.47	.62	1.74	.17

CES-D^a ^* TIME	2	0.46	.63	0.05	.95	0.92	.40	1.15	.32	2.74	.06	0.66	.52

CES-D^a ^* NRT * TIME	2	1.71	.18	0.02	.98	2.08	.13	0.67	.51	1.12	.32	2.22	.11

^a ^As a dichotomy in the model (CES-D > 15 = 'depressed'; CES-D ≤ 15 = 'not depressed'

#### Feeling anxious

In feeling anxious no NRT effects, but only a direct depression main effect (*F *= 4.8, *p *= .03) was found, suggesting that, on average, the depressed participants scored higher than the non-depressed participants, no matter the treatment condition was. (Figure 2) At those specific follow up points, there were no significant pair-wise differences.

#### Feeling sad

In feeling sad, there was a depression main effect (*F *= 9.8, *p *= .002), but also a NRT treatment effect (*F *= 4.5, *p *= .03). On average, the NRT groups reported lower sadness scores than the placebo groups. This was, generally speaking, slightly more visible among the depressed group, yet no significant depression by NRT interaction was found (*F *= 3.5, *p *= .06). (Figure 3) However, based on the Tukey HSD tests, none of the pair-wise differences at the specific time points were significant.

#### Feeling confused

In feeling confused we found a NRT main effect (*F *= 9.4, *p *= .002), indicating that, generally speaking, those in placebo treatment would score higher, a depression main effect, (*F *= 14.0, *p *< .001), suggesting that the depressed participants had higher scores than the non-depressed ones, and a significant depression by NRT interaction (*F *= 8.2, *p *= .005), suggesting that, on average, the treatment effect was more visible among the participants scoring high in the precessation depressive symptoms (Figure 4). According to Tukey HSD tests, pairwise differences at those specific time points were significant at 14 days only. The depressed placebo group had significantly higher mean score than the non-depressed placebo group (p = .005) and the non-depressed NRT group (p = .01). However, the depressed NRT group did not have significantly higher score than any of the non-depressed groups.

#### Feeling angry

In feeling angry we found a modest non-significant NRT main effect (*F *= 3.5, *p *= .06), a significant depression main effect, (*F *= 10.7, *p *= .001), but also a significant depression by NRT interaction (*F *= 5.5, *p *= .02), suggesting that, generally speaking, the treatment effect was more visible among the participants scoring high in the precessation depressive symptoms.

However, according to Tukey HSD tests, pair-wise differences at those specific time points were significant only at day 1(p = .04), and almost significant at day 7 (p = .08), the depressed in placebo group having higher mean score than the non-depressed in NRT and placebo groups, whereas the depressed in NRT group did not have significantly higher score than any of the non-depressed groups. A significant time effect (*F *= 4.2, *p *= .02) suggested that, on average, there were differences between the groups only at 1 and 7 days follow up points.

#### Feeling energetic and fatigue

As shown in Table 3, no significant NRT, baseline depression or time main effects or interactions were found in these energy-related mood states.

## Discussion

The present study evaluated the effect of Nicotine Replacement Therapy (NRT) on the post-quit mood profiles. The primary hypothesis tested whether pre-cessation sub-syndromal depression differentiated NRT's effects on Profile of Mood States (POMS), defined as six post cessation mood states. The results of the ANCOVA models suggested that pre-cessation depression modified NRT effects in some specific mood states. We found depression by NRT- interaction effects on feeling confused and feeling angry. On average, the depressed participants in the NRT condition had lower symptom scores than those depressed ones in the placebo group, such NRT effects being less visible among the non-depressed participants. In other words, the depressed participants in the placebo groups had the highest symptom scores. However, those depressed in NRT conditions did not have significantly higher symptom scores compared to the non-depressed groups, suggesting that NRT may be particularly important for those quitters scoring high in pre-cessation depression.

### Effects of NRT

In an earlier study Garvey et al. [24] found marginal effects of nicotine gum on self-reported post-cessation symptoms. There was a slight trend for the active gum users to report less increase in feeling confused compared to the placebo participants. However, the study did not include analyses by subgroups, such as by pre-cessation depressive symptoms. Thus, the present study was needed to give more insight into the specific effects of NRT among such sub-groups.

The form of NRT may play a role in the effects on the mood states. This trial used nicotine gum as a form of treatment. Using some other forms of NRT, such as nicotine patches, may not be as effective as nicotine gum in terms of some specific symptoms, such as mood effects. The nicotine patches, although being significantly effective in terms of abstinence, had only slight effects on measures of mood among the abstainers [23]. Further, the combination of nicotine patch and gum was found significantly more effective in alleviating withdrawal symptoms than any single treatment [10].

Also, the dose of NRT may play a role in those effects examined in our study. Initially, the participants were randomized to NRT (2 mg or 4 mg), or placebo groups. This report combined the two active treatment groups together. One may ask whether the groups differed in terms of dose, which might cause bias on the results. We analyzed this and found no biased effect caused by the nicotine dose in the active conditions among the depressed vs. non-depressed subgroups. First, 79% of the depressed participants, whereas 75% of the non-depressed were in the NRT condition (p = .52). Second, among those depressed in the NRT group 50% received 2 mg and 50% 4 mg dose of nicotine. Third, among those non-depressed 43.7% were in 2 mg and 56.3% in 4 mg condition (p = .44).

### Modifying Role of Depressive Symptoms

Our results that smokers with precessation depressive symptoms would be a particularly responsive to treatment of mood and energy related symptoms are supported by recent findings [30]. They suggested that one phenotype of tobacco dependence can be characterized by specific withdrawal symptomatology, which is actually primarily driven by depression. Although the POMS measures used in our study are not directly developed for measuring the withdrawal symptoms, they – if measured among the abstinent participants – can be used to track similar signs of withdrawal.

Different effects of NRT among depressed and non-depressed smokers have been explained also by genetic factors, such as by influence of the dopamine D4 receptor gene. It has been suggested that genetic factors involved in dopamine transmission may be associated with the beneficial effects of NRT for depressed smokers [31]. Thus, those more visible treatment effects for the depressed than for the non-depressed smokers would be mediated by dopamine transmission. This may provide an alternative explanation for our findings, although they were limited in two mood states only, i.e. feeling confused and feeling angry.

### Methodological Limitations

We analyzed the post-cessation mood profiles assuming that among the abstinent participants they represent similar signs as the withdrawal symptoms do after smoking cessation. We also assumed that these mood profiles are most relevant to be studied during the early period of abstinence. Our assumption is supported by Ward et al.[7] suggesting that the POMS would be identified as transient abstinence effects during the first month after cessation. Following these assumptions, we restricted our analyses among those participants only who were abstinent for at least two weeks.

These analyses concern a non-random sample of only those smokers who were able to achieve a continuous abstinence of two weeks. The procedure of including the abstainers only raises methodological concerns of possible bias between the original sample (n = 608) and the sub-sample used in our analyses (n = 242). The prevalence of the baseline depressive symptoms in the original sample was significantly higher than among the participants included in this secondary analysis. In other words, those with higher CES-D scores at baseline had already relapsed before two weeks and were not included in this analysis.

Actually, 43% of non-depressed and 32% of depressed participants survived through two weeks. Thus, it seems to be possible that the direct effect of the precessation depressive symptoms on post-cessation mood profile could have been somewhat diluted in the present study.

The drop-out analysis data in Table 1 also suggests that higher depression score, higher tobacco dependence as well as 'feeling sad' and 'fatigue' were, indeed, associated to relapse before two weeks. The observations that those with higher 'feeling sad' scores had relapsed before two weeks are consistent with previous results [5], where early relapse was associated with increased negative mood. However, our observations that the participants with highest depression scores had early relapse is contradictory with a recent meta-analysis suggesting that depression would not have direct effect on smoking cessation [13].

In this research paper the CES-D was used as a proxy for depression, rather than actual medical/psychiatric records or APA diagnostic criteria. Thus, we have to remember that the CES-D is indicative of sub-clinical depressive symptoms, but is not considered diagnostic of depression. A limitation of this study is that we may not have been able to address the question of whether NRT is particularly efficacious among individuals with major depressive disorder.

As a further limitation should be mentioned that the analysis of covariance (ANCOVA), which we used as the principal technique, assumes that the variable being analyzed is measured on a continuous interval scale and follows a normal distribution. Given that each of the POMS measures were 0 – 4 Likert scales, this may cause some concerns. However, ANCOVA is known to be very robust to departures from its assumptions.

### Clinical Significance

Whether or not NRT substantially reduces post-quit negative mood profiles more effectively for those with sub-syndromal depression has high clinical relevance. It is important to learn whether conventional treatment modalities effectively promote smoking cessation process among this subgroup of smokers. As mentioned earlier the data reported were collected on participants who remained abstinent at two weeks post-cessation. In order to appreciate the clinical significance of these results, it would be interesting to know if those mood states which appeared to be influenced by NRT and pre-cessation depressive symptoms were subsequently predictive of relapse.

Although beyond the scope of the present research question, we actually conducted a survival analysis on days abstained and found that a high 'Feeling confused' score predicted relapse (Hazard Ratio = 1.17; 95% CL = 1.01–1.35; p = .04). Also, a high 'Feeling sad' score was predictive for relapse ((Hazard Ratio = 1.13), although it did not reach statistical significance (95% CL = 0.98–1.30; p = .08). Although the evidence based on these analyses is not strong, another very recent study gives more support to the clinical significance of POMS in smoking cessation. Etter [32] found that at least some components of the mood profile, such as anxiety, depression, irritability (especially feeling angry) and difficulty concentrating (feeling confused) are associated to relapse.

Although not all our results achieved statistical significance, we would like to mention the possible clinical significance of them. For example, one clinically relevant finding is that in general, those smokers scoring high in pre-cessation depressive symptoms, if they don't receive any treatment for smoking cessation, may be suffering too much from adverse feelings during the first weeks of abstinence. Thus, in clinical practice, the level of depressive symptoms should be evaluated for every smoker before smoking cessation. If a patient expressed depressive symptoms and is willing to attempt quitting cigarettes, a clinician should consider suggesting antidepressant or NRT or combination treatments. Currently there is no clear evidence suggesting which antidepressant or form of NRT is the most effective for those with depressive symptoms [33,34]. Advantages of NRT include that it has minimal contraindications and it is available over-the-counter in most countries [2].

## Competing interests

The authors declare that they have no competing interests.
